# The induction of endoreduplication and polyploidy by elevated expression of 14-3-3γ

**DOI:** 10.18632/genesandcancer.161

**Published:** 2017-11

**Authors:** Cecil J. Gomes, Sara M. Centuori, Michael W. Harman, Charles W. Putnam, Charles W. Wolgemuth, Jesse D. Martinez

**Affiliations:** ^1^ University of Arizona Cancer Center, University of Arizona, Tucson, Arizona, USA; ^2^ Cancer Biology Graduate Interdisciplinary Program, University of Arizona, Tucson, Arizona, USA; ^3^ Department of Surgery, University of Arizona, Tucson, Arizona, USA; ^4^ Department of Physics, University of Arizona, Tucson, Arizona, USA; ^5^ Department of Molecular & Cellular Biology, University of Arizona, Tucson, Arizona, USA; ^6^ Department of Cell & Molecular Medicine, University of Arizona, Tucson, Arizona, USA; ^7^ Department of Surgical Research, Rhode Island Hospital, Providence, Rhode Island, USA; ^8^ Department of Engineering, Brown University, Providence, Rhode Island, USA

**Keywords:** 14-3-3 gamma, polyploidy, chromosomal instability, aneuploidy, non-small cell lung cancer

## Abstract

Several studies have demonstrated that specific 14-3-3 isoforms are frequently elevated in cancer and that these proteins play a role in human tumorigenesis. 14-3-3γ, an isoform recently demonstrated to function as an oncoprotein, is overexpressed in a variety of human cancers; however, its role in promoting tumorigenesis remains unclear. We previously reported that overexpression of 14-3-3γ caused the appearance of polyploid cells, a phenotype demonstrated to have profound tumor promoting properties. Here we examined the mechanism driving 14-3-3γ-induced polyploidization and the effect this has on genomic stability. Using FUCCI probes we showed that these polyploid cells appeared when diploid cells failed to enter mitosis and subsequently underwent endoreduplication. We then demonstrated that 14-3-3γ-induced polyploid cells experience significant chromosomal segregation errors during mitosis and observed that some of these cells stably propagate as tetraploids when isolated cells were expanded into stable cultures. These data lead us to conclude that overexpression of the 14-3-3γ promotes endoreduplication. We further investigated the role of 14-3-3γ in human NSCLC samples and found that its expression is significantly elevated in polyploid tumors. Collectively, these results suggests that 14-3-3γ may promote tumorigenesis through the production of a genetically unstable polyploid intermediate.

## INTRODUCTION

The 14-3-3 proteins are a family of ubiquitously expressed adaptor molecules that hold essential roles in the regulation of several physiological pathways. Limited with enzymatic activity [1], 14-3-3 proteins succeed in regulating a variety of mitogenic signaling pathways by modifying the functions of key proteins within said pathways. This is accomplished by a physical interaction between 14-3-3s and their target protein, typically mediated by a phospho-threonine or phospho-serine motif located on the target protein that is recognized by the 14-3-3 protein. The downstream effects facilitated by 14-3-3 binding include altering the enzymatic activity of the client protein, regulating subcellular localization, inhibiting protein-protein or protein-DNA interactions and protecting against dephosphorylation or proteolytic degradation [2, 3]. In mammals, there are seven isoforms encoded by seven distinct genes (β, γ, ε, η, σ, θ, and ζ), and between them over 200 known binding partners [4]; illustrating why these proteins function in such a diverse array of cellular processes.

Perhaps the most notable role for 14-3-3 proteins is their extensive regulation of cell cycle progression [5]; they act at several junctures of the G1/S and G2/M transitions to prevent premature succession until the cell has met the demands of the checkpoint requirements. However, these proteins are not limited to cell cycle regulation and are involved in a variety of cellular processes including cellular growth, survival and migration (a comprehensive overview of 14-3-3 proteins can be found at [6]). Hence, it is not surprising that abnormal 14-3-3 expression patterns have been linked to human tumorigenesis [7].

In human tissues, 14-3-3 proteins have been observed to function as tumor promoters and tumor suppressors when aberrantly expressed [4, 8], with each role appearing isoform and tissue specific. For instance, several studies have demonstrated that 14-3-3σ (also known as *stratifin or HME1*) functions to suppress tumor formation in breast tissue and that its expression becomes significantly reduced by hypermethylation of its promoter region during tumorigenesis [9–11]. In contrast, an isoform with the capacity to function as an oncoprotein, 14-3-3γ, becomes significantly upregulated in a variety of human cancers and has been characterized as a prognostic marker for poorer survival in breast, lung and hepatocellular carcinomas [12–15]. Unlike the tumor suppressing properties of 14-3-3σ, which have been extensively outlined [16], the mechanism(s) driving 14-3-3γ's tumor promoting effects remain unclear. Nevertheless, the diverging phenotypes between σ and γ have been proposed to originate from relatively few differences in amino acid sequence located within variable regions of the N-terminus for both isoforms [17]. Our laboratory is interested in defining the oncogenic phenotype that manifests with increased expression of 14-3-3γ.

In previous attempts to elucidate 14-3-3γ's role in promoting tumorigenicity, our group demonstrated that overexpression of this isoform resulted in neoplastic transformation of NIH-3T3 cells, and through mutational studies we were further able to show that transformation was dependent upon activation of the Ras/Raf MAP kinase and PI3 Kinase signaling pathways [15]. In addition to the oncogenic properties promoted by 14-3-3γ overexpression, it was also observed that a sub-population of cells exhibited polyploid, a phenotype derived from whole genome duplication events and characterized by having multiple sets of homologous chromosomes. Polyploidy was again observed subsequent to overexpression of 14-3-3γ in a lung cancer and leukemia derived cell line [18], indicating that abnormal cell cycle progression and polyploidization may be a common effect from elevated levels of this 14-3-3 isoform.

Several studies have demonstrated that polyploid tumor cells adapt and evolve more rapidly than their diploid counterparts; their elevated rates of genomic instability promotes adaptation and evolution while their inherent increase in ploidy buffers the lethality of deleterious mutations [19–22]. Moreover, polyploid cells are distinctively resistant to radio and chemotherapies [23], suggesting that polyploid cells remain key players driving the ongoing evolution of patient disease. In this study, we characterized the mechanism driving 14-3-3γ-induced polyploidization and the effects that this has on genomic stability. We also addressed the relationship between elevated expression of 14-3-3γ and the frequency of polyploidy observed in lung cancers.

## RESULTS

### Elevated levels of 14-3-3γ increases the incidence of polyploidy approximately four fold

**Figure 1:**
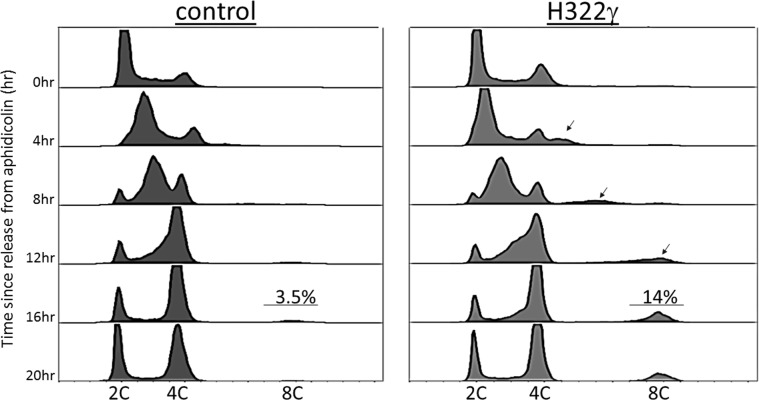
Overexpression of 14-3-3γ induces polyploidy H322 cells mock transfected with an empty vector (control) or with 14-3-3γ (H322γ) were synchronized at the G1/S transition with aphidicolin, released into complete media and harvested at 4 hour intervals for cell cycle analysis by flow cytometry. Histograms of events collected are shown and the frequency of polyploid cells are presented at the 16 hour time-point for both control (left) and H322γ (right) populations. The origins of polyploid H322γ cells are shown (black arrows). On the x-axis is DNA content and on the y-axis time since release from Aphidicolin.

Previous research in our laboratory has demonstrated that overexpression of 14-3-3γ results in the production of polyploidy [24, 17]. To investigate the proportion of polyploid cells induced by 14-3-3γ overexpression, we quantitated the frequency of polyploidy in H322γ cells, a lung cancer-derived cell line transfected with an expression vector that constitutively expresses the 14-3-3γ gene (*YWHAG*). An increase in 14-3-3γ protein expression was confirmed by western blot (data not shown). H322γ and parental cells that were transfected with an empty vector (referred to simply as control) were synchronized at the G1/S transition with treatment of Aphidicolin for 24 hours, at which point they were released into complete media and collected at regular intervals for cell cycle analysis by flow cytometry. As seen in Figure 1, H322γ cells display a distinct peak of polyploid cells (black arrows) from 4-20 hours post synchronization, which constitutes 14% of the total population. Polyploid cells were also present in the control cells, but constituted only 3.5% of the population, confirming that elevated levels of 14-3-3γ induces a four-fold increase in the incidence of polyploidy.

### 14-3-3γ overexpression results in mononucleated polyploid cells

**Figure 2:**
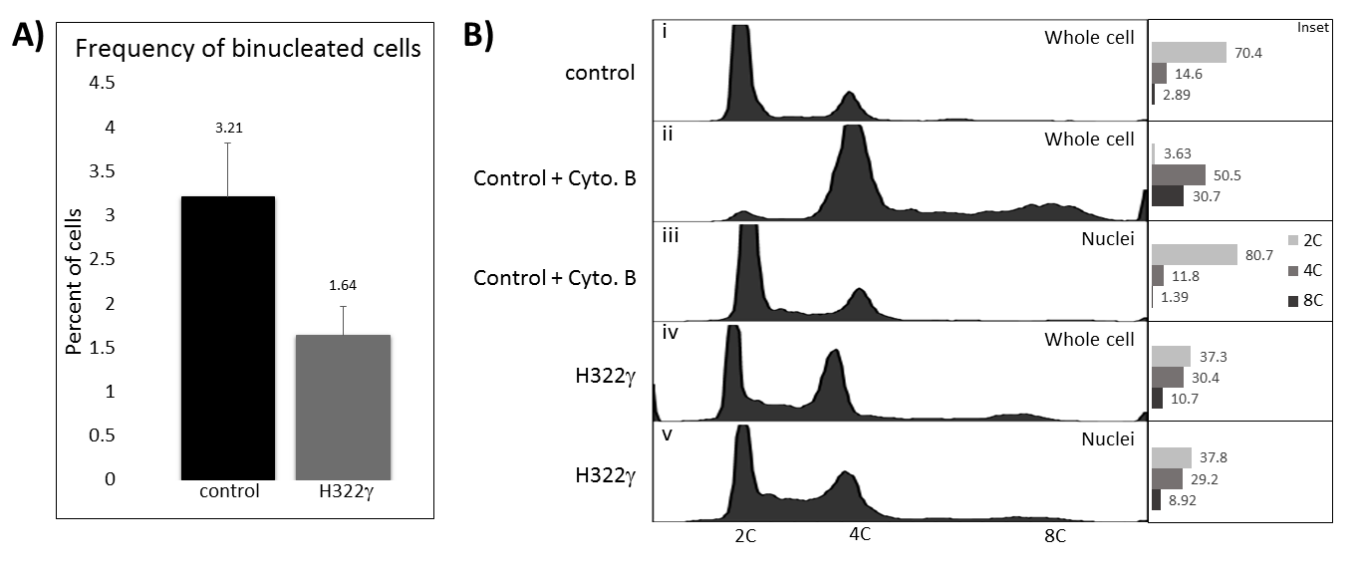
Overexpression of 14-3-3γ leads to mononucleated polyploid cells A) The percentage of binucleation within control (black bar) and H322γ cells (grey bar) are presented as a bar graph, which represents the frequency of binucleated cells found within 500 cells scored per group. This experiment was repeated three times and the average for each population is presented. B) Control cells remained untreated (i), treated with cytochalasin B (Cyto. B) for 24 hours (ii) or treated with cytochalasin B followed by nuclear fractionation (iii). H322γ cells remained untreated (iv) or underwent nuclear fractionation (v). Flow cytometry was performed on whole cells and isolated nuclei from both control and H322γ cells. On the x-axis is DNA content and on the y-axis histogram count. Inset: The proportion of cells with 2C, 4C and 8C DNA content are presented as bar graphs. This experiment was performed three times and representative examples are displayed.

While imaging H322γ and control cells, we noted that binucleated cells appeared more often in the control population than in H322γ cells. This prompted us to investigate whether the polyploid cells observed within the H322γ population were phenotypically similar to the polyploid cells that exist at lower levels in the control population. To do this, we first determined the prevalence of binucleated cells in H322γ cells versus the vector-only control cells. The incidence of single cells harboring two nuclei existed at approximately 3.2% within the control population, nearly identical to the incidence of polyploidy observed in Figure 1 for this population. However, approximately 1.6% of H322γ cells were binucleated, accounting for only a fraction of the polyploidy produced in H322γ cells, suggesting that 14-3-3γ overexpression is promoting an alternative route to polyploidy from what is observed in the control cells (Figure 2A).

To further substantiate the latter finding, we used an approach that compares cellular ploidy before and after dissolution of the cell membrane to obtain cell cycle profiles on isolated nuclei. To validate the methodology, H322 cells were treated with cytochalasian B, a mycotoxin that strongly inhibits cytokinesis, producing a substantial binucleate population when compared to untreated cells (8C, 2.9% and 30.7%, respectively), Figure 2i & 2ii; isolated nuclei from the same cell population however were, as predicted, nearly all euploid (8C, 1.4%), Figure 2iii. Hence, the polyploid peaks populated from binucleated cells vanish when the cellular membranes are dissolved. In contrast, whole cells and isolated nuclei from the H322γ population had similar rates of polyploidy (10.7% and 8.92%, respectively), Figure 2iv & 2v.

Note that there is a slight reduction in the frequency of 8C cells within the H322γ population treated with the nuclear isolation buffer, accounting for a reduction by approximately 1.8%, which is likely due to the small number of binucleated cells seen in these cultures (Figure 2A). Taken together, this data indicates that overexpression of 14-3-3γ is promoting mononucleated polyploidization.

### Overexpression of 14-3-3γ promotes endoreduplication

**Figure 3:**
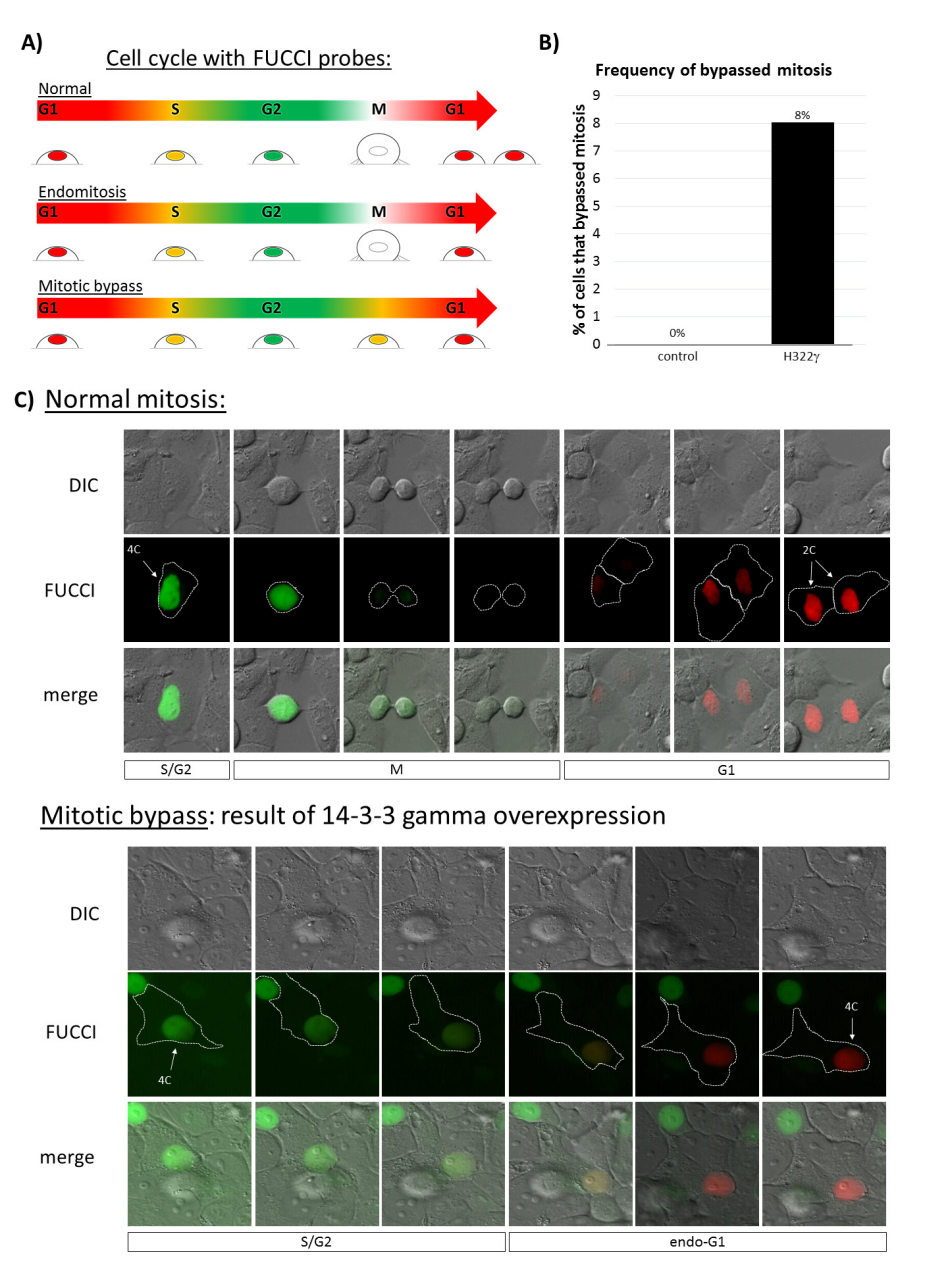
Overexpression of 14-3-3γ promotes mitotic bypass A) The changes in nuclear fluorescence among cells expressing the FUCCI probes are diagrammed as arrows. Reading from left to right, the stages of the cell cycle are labeled within each arrow and the changes in the color of the cells are shown underneath. Methodically, the changes in cell surface adhesion throughout each stage of the cell cycle is also depicted. The top arrow depicts the color changes observed in cells progressing through a normal cell cycle. The middle arrow displays the endomitotic process and the bottom arrow the process of mitotic bypass. B) Control and H322γ cells transduced with the FUCCI dual reporter system were tracked through time by live-cell fluorescence microscopy. Control cells (n = 78) H322γ cells (n = 158) were manually tracked for normal mitotic progression, endomitosis and mitotic bypass. Mitotic bypass was the only error detected, therefore the incidence observed in control and H322γ cells are presented. The experiment was performed twice and the bars on the graph show the average for all of the cells that were counted in the two experiments. C) Representative examples from panel B are shown. Time-lapse images of live cells using differential interference contrast (DIC) microscopy and epiflouresence (FUCCI) are presented, with the cell border outlined in white. The merged images (merge) are also shown and the stages of the cell cycle are labeled underneath each set of images. The upper set of images presents a control cell progressing through a normal mitosis. The bottom set of images is of a H322γ cell progressing through the cell cycle in which mitosis has been bypassed, leading to endoreduplication. Note that the two distinguishing features of mitotic bypass is the lack of a cell cycle stage where there is no fluorescence in the nucleus between a green to red transition, while also no evident changes in cell surface adherence, as would be observed in a canonical progression through mitosis, indicating that mitosis has been skipped.

Having established that the polyploid phenotype occasioned by the overexpression of 14-3-3γ is mononucleate, we sought to identify the mechanism by which polyploidy is induced. Mononucleated polyploidization from a diploid progenitor can occur in two ways – (i) endoreduplicaiton, which ensues when diploid cells in G2 suppress the requirements necessary for entry into mitosis, allowing cells to bypass mitosis entirely and enter a polyploid or endo-G1 state. These cells either continue endocycling - alternating between G and S phases to further increase ploidy - or more commonly, return to a canonical cell cycle and propagate as polyploid cells. (ii) Endomitosis, which occurs when cells fail to complete nuclear segregation in anaphase, leading to premature exit from mitosis [25]. To determine the mechanism promoting mononucleated polyploidization in H322γ cells, we performed live-cell microscopy in combination with the FUCCI system (fluorescent ubiquitination cell cycle indicators). The FUCCI probes are a dual reporter system that allows tracking of live cells through the cell cycle and has been demonstrated to accurately detect cells undergoing endoreduplication or endomitosis [26, 27]. The key distinction between endomitosis and endoreduplication is the complete absence of mitosis in cells undergoing endoreduplication before entering a polyploid G1 state, which we have diagramed in Figure 3A. We examined FUCCI expressing H322γ cells using live cell microscopy and found no evidence that H322γ cells underwent endomitosis. Instead, overexpression of 14-3-3γ resulted in 8% of cells bypassing entry into mitosis (Figure 3B). Representative examples of control cells undergoing normal mitotic progression and gamma cells bypassing mitosis are presented (Figure 3C). This data is consistent with the notion that 14-3-3γ acts to negatively regulate mitotic entry [5] and that overexpression suppresses the onset of mitosis, a hallmark of endoreduplication.

### Both diploid and polyploid cells overexpressing 14-3-3γ exhibit prolonged mitoses accompanied by chromosomal segregation errors

In addition to enumerating cells undergoing mitotic bypass, data compiled from H322 cells overexpressing 14-3-3γ using the FUCCI system also suggested that M-phase was often prolonged in those cells successfully negotiating mitosis. To accurately measure the duration of mitosis and its component prophase to metaphase and metaphase to anaphase transitions, control and H322γ cells were transfected with a GFP construct of histone H2B, which enables imaging of chromosomes. Time-lapse video microscopy was performed on control and H322γ cells that were co-transfected with pBOS-H2B-GFP and imaged consecutively for 18 hours at 5 minute intervals between acquisitions. Shortly before the conclusion of the image acquisition sequence, Hoechst 33342 was added to the culture medium, in order to quantify DNA content in each cell (see Methods); it was then possible to retrospectively identify a polyploid versus diploid progenitor cell based upon the summation of the DNA contents of its daughter cells. Thus each cell tracked through mitosis could be labeled as initially diploid or polyploid.

**Figure 4:**
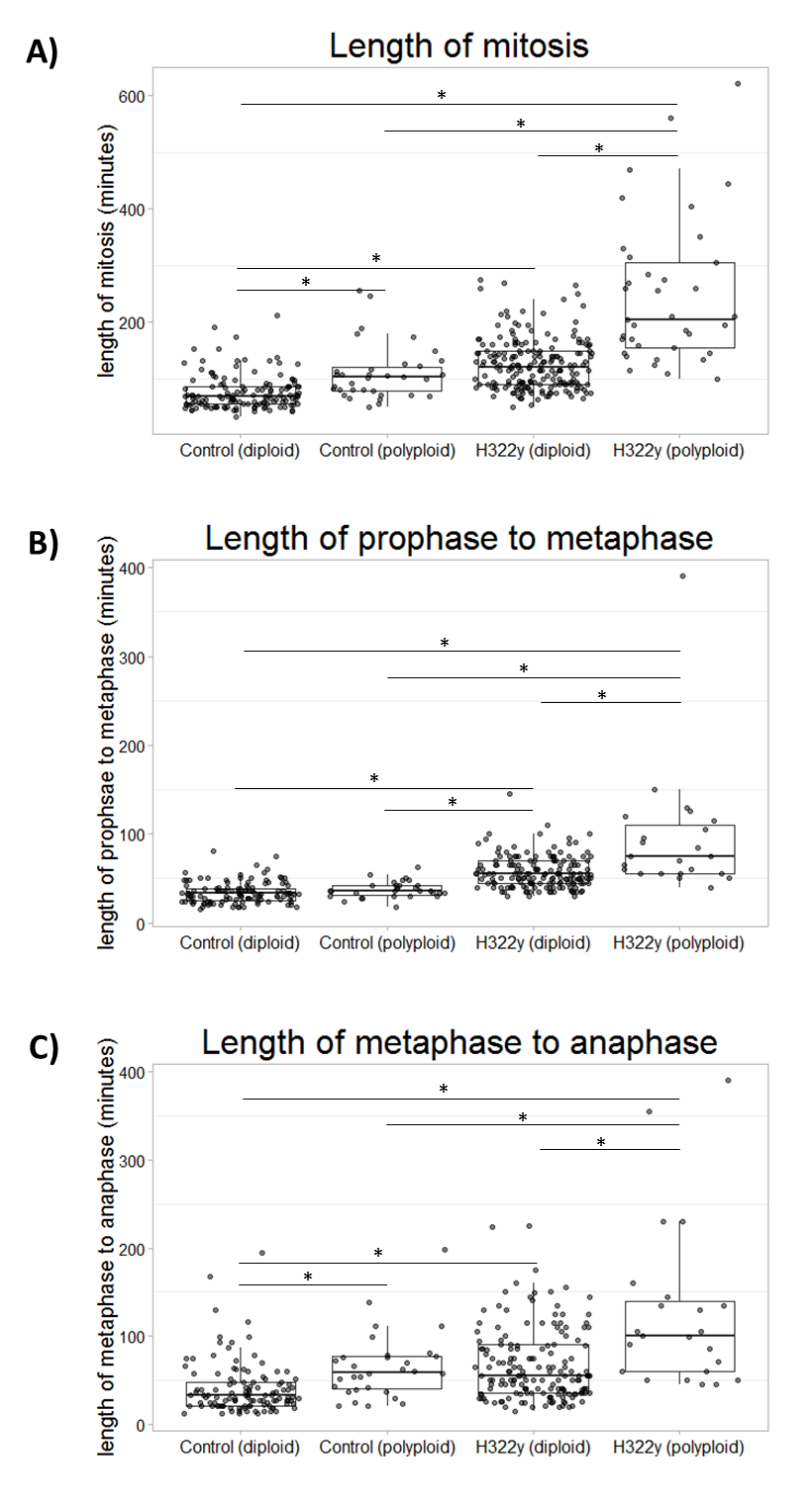
14-3-3γ derived mononucleated tetraploid cells have a significantly prolonged mitoses H322γ and control cells were co-transfected with a vector driving the expression of H2B-GFP. Asynchronous control-H2B-GFP cells and H322γ-H2B-GFP cells were imaged using time lapse microscopy over a period of 16 hours with images taken at 3 minute intervals for the length of the experiment. Both DIC and epifluorescence images were acquired. The live-cell DNA dye, Hoechst 33342, was added at the end of the experiment to allow for DNA content analysis. The ploidy of each mitotic cell was calculated based on combining the nuclear Hoechst 33342 fluorescence of each daughter cell. A) The length of mitosis, measured from nuclear envelope break down to the beginning of anaphase, is presented as a box plot for diploid control cells (n=134), polyploid control cells (n=34), diploid H322γ cells (n=197) and polyploid H322γ cells (n=37). Each dot represents the length of mitosis for a single cell within the appropriate cohort. B) A boxplot depicts the length of prophase to metaphase and C) the length of metaphase to anaphase. This experiment was repeated two times. A Welch's t-test was performed and statistical significance was measured at p < 0.05, indicated by an asterisk.

As can be seen in Figure 4A, polyploid H322γ cells experience significantly prolonged mitoses when compared to diploid H322γ cells. To determine if this lengthening effect was ploidy dependent, polyploid H322γ cells were compared against the binucleated polyploid cells observed within the control population (referred to as control polyploid cells). We found that the polyploid H322γ cells proceeded through mitosis at a delayed rate when compared to the control polyploid cells, suggesting that overexpression of 14-3-3γ interferes with mitotic progression. Consistent with these results, diploid H322γ cells also experienced prolonged mitoses when compared to diploid control cells, strengthening the notion that 14-3-3γ has a role in mitotic progression. Notably, the gamma lengthening effect also resulted in an increase in the duration of intermediate steps, prophase to metaphase and metaphase to anaphase (Figure 4B & 4C). It is important to note that in each step evaluated the polyploid H322γ population had the largest delay when compared to all other groups.

**Figure 5:**
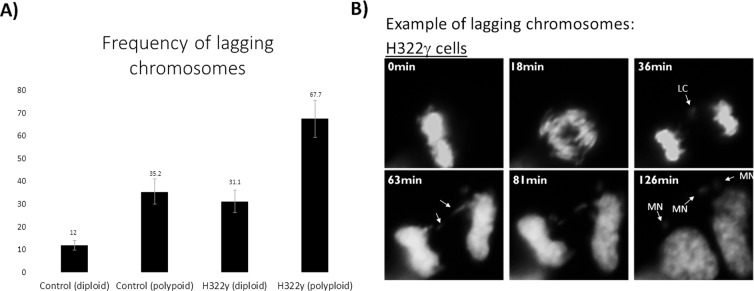
Overexpression of 14-3-3γ promotes chromosomal instability Time lapse microscopy was performed on H322γ and control cells expressing H2B-GFP. Individual cells were manually tracked as they progressed though mitosis and the percent of cells with lagging chromosomes in anaphase were documented. A) The frequency of lagging chromosomes within each group examined are presented for as a bar graph. This experiment was repeated two times with the average for each group presented above error bars, which depict upper and lower limits between the two experiments. B) Representative example of lagging chromosomes (LC) and subsequent micronuclei (MN) observed in the H322γ population are displayed. The images illustrate mitotic progression from metaphase through the completion of mitosis with the elapsed time between acquisitions displayed in the top left corner of each image.

Delays in mitotic progression often result from chromosomal segregation errors [28, 29], therefore we examined if this relationship remained consistent in polyploid H322γ cells. By tracking the movement of chromosomes in mitotic cells as they transitioned from metaphase to anaphase, we observed that the polyploid H322γ population experienced a marked increase in lagging chromosomes when compared to diploid H322γ cells and control cells, both diploid and polyploid (Figure 5A). Interestingly, diploid H322γ cells also presented with an increased incidence of lagging chromosomes when compared to diploid control cells, suggesting that 14-3-3γ overexpression may be sufficient to disrupt proper chromosome segregation in mitosis. The majority of these lagging chromosomes failed to merge into the daughter nuclei of nascent cells and subsequently resulted in micronuclei (Figure 5B). Taken together, these results demonstrate that elevated levels of 14-3-3γ resulted in a prolonged and error-prone mitosis, and that this phenotype is greatly exacerbated in polyploid cells.]

### 14-3-3γ-overexpressing tetraploid cells perpetuate over time

Polyploid cells have an inherent capacity to adapt and evolve at accelerated rates compared to diploid counterparts [20,21,30]; this has led to the suggestion that in tumors, polyploid cells represent intermediaries which give rise to aneuploid cells by virtue of chromosomal instability [31]. Our observation that the incidence of lagging chromosomes and micronuclei is increased in polyploid H322γ cells is consonant with this hypothesis. Consequently, we sought evidence of the possible influence of 14-3-3γ overexpression on the genomic evolution of polyploid cells. We successfully isolated and expanded approximately 20 polyploid cells from both control and H322γ cell cultures that were sorted by flow cytometry into individual wells of 96-well plates and the resulting colonies progressively expanded (Supplementary Figure 1). Polyploidy was verified using flow cytometry once the clones had successfully expanded into 6-well culture dishes.

**Figure 6:**
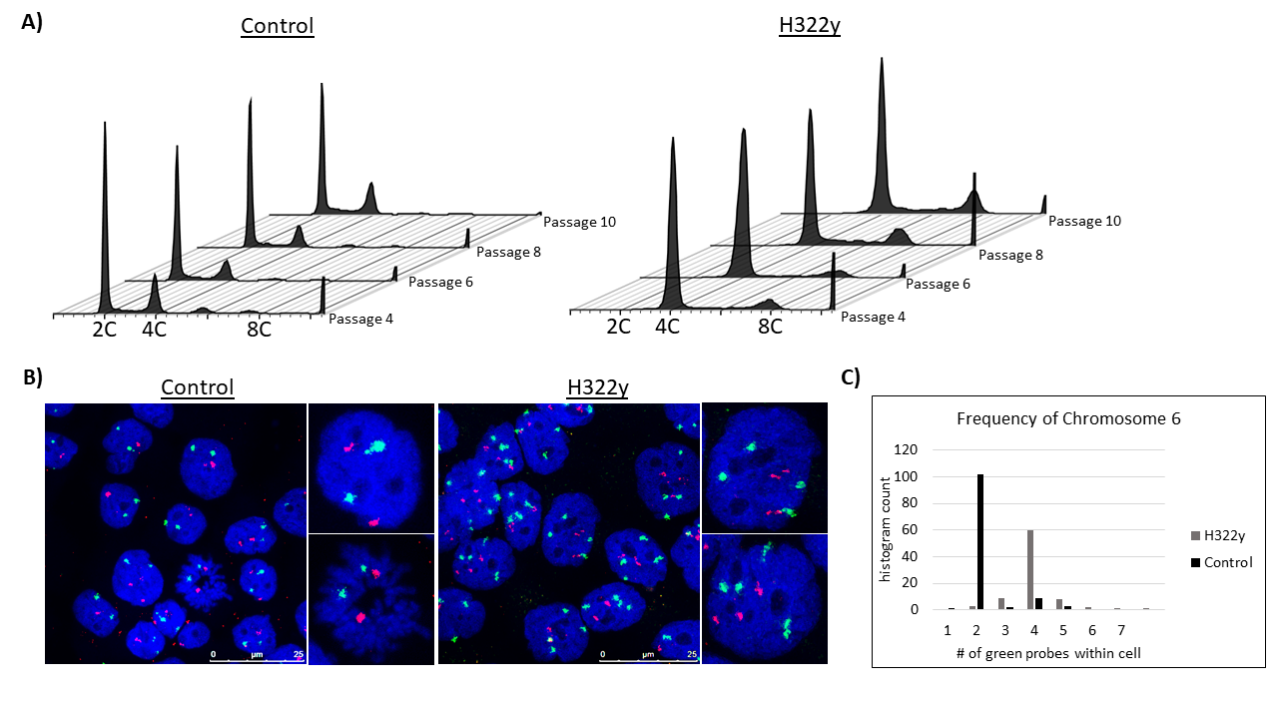
14-3-3γ-overexpressing tetraploid cells perpetuate over time Control and H322γ cells were stained with Hoechst 33342 and FACS sorted. Single cells were seeded per well and the resulting colonies expanded. Approximately 20 clones from each group were grown in culture and passaged for minimally 10 iterations. Representative samples were saved at each passage and analyzed by flow cytometry under identical conditions for each passage. A) Representative flow cytometry histograms are shown for both control and H322γ clones, with passage number on the z-axis and DNA content on the x-axis. B) Numerical quantification of chromosome copy numbers were assessed at passage 10 using FISH against the centromeric regions of chromosomes 6 (green) and 18 (red), DAPI in blue. Representative images are displayed. C) The modal chromosome counts for chromosome 6 are displayed as a histogram.

We propagated the clones for an extended period of time and examined their ploidy at regular intervals using flow cytometry and tested for chromosome numerical abnormalities using fluorescence *in situ* hybridization (FISH) probes against the centromeric regions of chromosomes 6 and 18. We found that all of the spontaneous tetraploid clones isolated from the control population quickly reverted to a diploid or near-diploid karyotype by passage three, Figure 6A. In contrast, despite being maintained under identical conditions, 20 of the 14-3-3γ-overexpressing tetraploid clones continued to exhibit elevated genomic ploidy for at least 10 passages. Only one of the polyploid clones isolated from the H322γ population reverted to a near-diploid karyotype before reaching passage 10. FISH was employed at passage 10 to further demonstrate the numerical differences between clones isolated from the control cells versus those from the H322γ population. Representative examples are presented in Figure 6B. Quantitation of the modal copy number of chromosome 6 in both the control group (modal = 2) and H322γ cells (modal = 4) confirms a stable tetraploid genome in polyploid clones isolated from H322γ cells, Figure 6C. Hence, 14-3-3γ overexpression predisposes cells toward having an elevated DNA content that is stable over time.

### Elevated levels of 14-3-3γ correlate with polyploid NSCLCs *in vivo*

**Figure 7:**
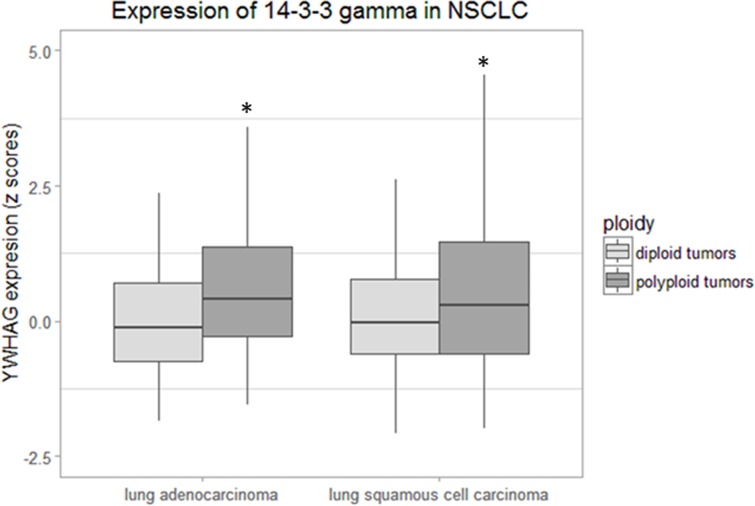
14-3-3γ mRNA expression is elevated in lung samples predicted to be genome doubled A Welch's t-test was performed and statistical significance was measured at p < 0.05, indicated by an asterisk. [LUAD = lung adenocarcinoma (n=257), LUSC = lung squamous cell carcinoma (n=138)].

The data presented in the preceding sections document that in the H322 non-small cell lung carcinoma cell line, overexpression of YWHAG, resulting in an excess of the 14-3-3γ protein, increases the prevalence of mononucleate polyploidy by endoreduplication, predisposes to chromosome segregation errors, and promotes a stable polyploid phenotype. Polyploidy has been documented in a variety of human cancers; an incidence of 36 - 47% has been estimated in NSCLC [20, 31]. Hence, we sought to test the prediction that human tumors in which polyploidy is present will exhibit an increased expression of 14-3-3γ. To do so, we utilized data compiled from lung adenocarcinomas (LUAD) and lung squamous cell carcinomas (LUSC) archived in *The Cancer Genome Atlas* (TCGA). SNP6.0 data were analyzed, as described by Dewhurst *et al.* [20], as a measure of ploidy (see Methods). Expression values of YWHAG, the 14-3-3γ gene, were gathered as z-scores (see Methods), to obviate differences in overall gene expression levels between samples. Following this procedure, mRNA z-score expression values for the 14-3-3γ gene were compared across samples predicted to be either diploid or polyploid. Interestingly, 14-3-3γ was significantly elevated in samples estimated to be polyploid in both lung adenocarcinoma and squamous cell carcinoma samples indicating that 14-3-3γ expression positively correlates with the incidence of polyploidy (Figure 7). A similar relationship between YWHAG expression and polyploidy was also found when colorectal or breast adenocarcinoma data from TCGA were analyzed in the same fashion (Supplementary Figure 2), suggesting that the relationship between upregulation of 14-3-3γ and polyploidy is not specific to lung cancers. Taken together, these data support our hypothesis that overexpression of YWHAG and the consequent excess of the 14-3-3γ protein contribute to the polyploidy frequently observed in human NSCLC and other carcinomas.

## DISCUSSION

14-3-3γ is a recognized oncoprotein that is overexpressed in human lung cancers [36] and has been characterized as a prognostic marker for poorer survival in patients with advanced disease [12], indicating that 14-3-3γ plays a role in promoting lung tumorigenesis. Here we examined the role that overexpression of 14-3-3γ has on cell division and found that overexpression promotes the development of cells with abnormal numbers of chromosomes. Lung cancer cells transfected with 14-3-3γ have a readily detectable fraction of cells with 8C DNA content. By combining time-lapse video microscopy with the FUCCI dual probe system we were able to show that the transition from G2 to M phase is compromised in H322γ cells, forcing them to bypass mitosis and reenter growth phase I with double the normal DNA content. Notably, the modal chromosome number observed in these cells is a doubling of the full complement of chromosomes with intermediate quantities of DNA content observed only in cells undergoing DNA replication during S phase. These observations are best explained by the concept that 14-3-3γ acts primarily at the point of entry into mitosis but does not interfere with reestablishment of the G1 phase and relicensing of DNA for replication in a subsequent S phase. This is consistent with what has been observed with 14-3-3σ when overexpressed in colorectal cancer cells [33], suggesting that regulation of entry into mitosis may be a common function to 14-3-3 proteins and polyploidy is a likely result when levels of these proteins are elevated.

Although we have demonstrated that 14-3-3 proteins influence mitotic entry, the mechanism of how this occurs remains unclear. It is well documented that cyclin-dependent kinase (CDK) activity is what drives the G2 to M phase transition and is believed that CDK activity must meet a threshold before a cell can enter M phase [34, 35]; we have previously demonstrated that CDK activity is significantly reduced in cells overexpressing 14-3-3γ approaching the G2 to M phase transition [18]. To prevent premature entry into mitosis, Cdk1 is maintained in an inhibited state by phosphorylation of T14 and Y15 within the ATP-binding “P-loop” by kinases Wee1 and Myt1 [36]. It is the removal of these inhibitory P-loop phosphate groups on Cdk1 by the Cdc25 family of phosphatases that permit CDK activity to reach a mitotic threshold sufficient to enter mitosis [37, 38]. 14-3-3γ has been established to act directly on these Cdk1 regulators. Direct 14-3-3 binding to kinases Wee1 and Myt1 has been demonstrated to significantly enhance the inhibition of Cdk1 [36, 39]. 14-3-3 binding to phosphatases Cdc25B/C results in a direct reduction in enzymatic activity [40, 41] and cytoplasmic sequestration of these proteins, thereby disallowing access to its nuclear substrate Cdk1/cyclin B [42–44]. Moreover, co-IP experiments overexpressing tagged 14-3-3 isoforms showed that ε and γ have the strongest binding affinity to Cdc25C *in vitro* [45], however, a mutation on Cdc25C at Ser216/287 to a non-phosphorylatable residue disrupts 14-3-3 binding and thereby inhibits nuclear export [46]. To this end, depletion of 14-3-3 gamma by shRNA results in a compromised G2 checkpoint allowing cells enter mitosis prematurely [47], likely a result of the inability to inhibit Cdk1 [46]. Presumably, elevated expression of 14-3-3γ, a key negative regulator of the G2 to M phase transition, acts on one or more upstream regulators of Cdk1 as describe above.

However, it is unclear the requirements necessary for a cell to bypass mitosis entirely. For instance, how long cells must arrest at the G2/M interface before they commit to bypassing mitosis or whether CDK activity must meet a lower-bound threshold before cells commit to skipping mitosis remains unresolved. Unfortunately, the FUCCI system is incapable of differentiating DNA synthesis and growth phase II of the cell cycle, disallowing the ability to quantify the amount of time H322γ cells arrested in G2 before they skipped mitosis. This area of research warrants further investigation to thoroughly understand the mechanism driving mitotic bypass.

Interestingly, H322γ cells that do undergo mitosis xhibit a prolonged mitotic phase, even when compared to the length of mitosis in polyploid cells observed within the control population. 14-3-3γ overexpression results in nearly a doubling in the duration of mitosis. Moreover, progression through the intermediate mitotic steps are also prolonged suggesting that 14-3-3γ may have an impact on more than just mitotic entry. It has been demonstrated that knockdown of 14-3-3γ also induces arrest in mitosis due to activation of the spindle assembly checkpoint, suggesting that substantial changes in 14-3-3γ expression may be sufficient to perturb normal mitotic progression [48]. Perhaps defects in chromosome segregation are the cause of this lengthening effect, as H322γ cells also experienced an increased frequency of lagging chromosomes during anaphase of mitosis. Interestingly, this increase in chromosomal segregation errors did not correlate with an increase in centrosome copy number, which was assessed by immunofluorescence (data not included). Taken together, both the length of mitosis and the frequency of chromosomal segregation errors were significantly increased in polyploid H322γ cells, suggesting that 14-3-3γ-derived polyploid cells are particularly unstable.

Hence, it was surprising to find that purified polyploid H322γ cells maintained a stable chromosome number under prolonged propagation in culture. Compared to polyploid control cells that revert to a diploid content by passage three, polyploid H322γ cells remained polyploid for the duration of the experiment. In retrospect, none of our previous studies showed aneuploidy in cells that overexpress 14-3-3γ [18]. However, we consistently observed polyploidy. It is notable that polyploidy was consistently associated with transformation of rodent cells [15]. This suggests that polyploidy by itself may contribute to tumorigenesis. Collectively, our data indicates that 14-3-3γ can induce polyploidy but that the development of aneuploidy may require additional mutations which further destabilizes chromosome segregation.

In order to investigate the relationship between 14-3-3γ expression and polyploidy observed in human lung cancers we examined lung cancers in the TCGA data set. We found that 14-3-3γ is significantly elevated in samples predicted to be polyploid, indicating that 14-3-3γ may have a prominent role in promoting polyploidization in lung tumors. Polyploidy is an independently predictive of poor relapse free survival [20], suggesting that polyploidy may promote tumorigenesis, which is consistent with our in vitro studies which relates 14-3-3γ oncogenic function, polyploidy, and transformation [15].

Taken together, our studies suggest that 14-3-3γ may play a role in tumorigenesis by inducing polyploidy; a phenotype that, by itself, may promote tumorigenesis and set the stage for further changes that lead to neoplastic progression.

## MATERIAL AND METHODS

### Plasmids

The empty pCMV-Tag2 vector and pCMV-Tag2-YWHAG (sub-cloned 14-3-3γ) vector were obtained from Dr. Radhakrishnan, University of Arizona, AZ, USA. The pBOS-H2B-GFP vector was obtained from Dr. Wahl, Salk Institute for Biological Studies, La Jolla, CA, USA.

### Cell culture and transfections

H322 cells were purchased from the American Type Culture Collection (Rockville, MD, USA). Cells were maintained in Dulbecco's modified Eagle's medium (Corning Cellgrow, Manassas, VA, USA) supplemented with 10% fetal bovine serum (Peak Serum, Fort Collins, CO), 100 U penicillin and 100 mg streptomycin and maintained at 37°C in a humidified atmosphere of 5% CO_2_. Cells were transfected using Lipofectamine LTX with PLUS reagent (Invitrogen, Carlsbad, CA, USA). Cells expressing the pCMV-tag2 vector were maintained in 0.5 mg/ml G418 (Invitrogen, Carlsbad, CA, USA). Cells transfected with pBOS-H2B-GFP were selected with 3ug/ml Blastcidin S HCl (Sigma-aldrich, St. Louis, MO, USA) and FACS sorted by GFP expression. These cells were then maintained with 1 ug/ml Blasticidin S HCl in the culture medium. H322 cells were authenticated at the University of Arizona Genetics Core by autosomal STR profiling in May of 2017.

### Cell synchronization

Logarithmically growing cells were plated and allowed to adhere for 24 hours before adding 3ug/ml aphidicolin (Sigma-aldrich, St. Louis, MO, USA) or DMSO (Sigma-aldrich, St. Louis, MO, USA) for an additional 24 hours. Following synchronization, cells were washed with 1X DPBS (Corning, Manassas, VA, USA) before being returned into 10% complete media. Cells were harvested at various time-points following release from synchronization.

## FACS

Assessment of DNA content was carried out using a BD FACScanto II flow cytometer (BD Biosciences, San Jose, CA, USA) and cell cycle histograms generated using the FlowJo V10 (Ashland, Oregon) software package. Cells were fixed by drop-wise addition of 70% ice-cold ethanol while vortexing. Samples were then treated with RNAse A (Sigma-aldrich, St. Louis, MO, USA), stained with Propidium iodide (Sigma-aldrich, St. Louis, MO, USA) and incubated at 37°C for 30 minutes prior to cytometric analysis. Cell aggregates were gated out of the analysis, determined by PI-A and PI-W. To assess DNA content in fractionated nuclei, cells were pelleted and suspended in nuclei isolation buffer I (584 mg/L NaCl, 1000 mg/L Na-citrate, 10 mg/l RNase A, 0.3 mL/L Nonidet P40 and 0.05 mg/ml propidium iodide) and stored at 4°C in the dark for 1 hour. Equal volume of nuclei isolation buffer II (15 g/L cirtic acid, 0.25 M sucrose, 0.05 mg/ml propidium iodide) was then added and samples were stored at 4°C until cytometric analysis, no later than three days from collection. In cell sorting experiments a FACS Aria III (BD Biosciences, San Jose, CA, USA) cell sorter was used to sort single cells stained with Hoechst 33342 (Thermofisher Scientific, Waltham, MA, USA) at 2ug/ml into 96 well plates supplemented with 20% FBS media. Successful colonies were progressively expanded into 10 cm dishes and passaged approximately once a week (80% confluence) at a consistent 1/5 dilution.

## FISH

Cells were plated into coverglass bottom removable chamber slides (Thermofisher Scientific, Waltham, MA, USA) and allowed to adhere for 24 hours at 37°C and 5% CO_2_. The cells were then fixed in 3:1 methanol/acetic-acid. The slides were then denatured, hybridized with XCE probes 6-green and 18-orange, and washed according to the FISH protocol for MetaSystems' DNA probes provided by Metasystems. Following hybridization, the slides were counterstained with prolong DAPI-antifade gold mounting medium (Invitrogen, Carlsbad, CA, USA) and imaged with a Leica 5-confocal microscope (Leica Microsystems Inc., Buffalo Grove, IL, USA). Slides were stored in the dark at -20°C.

### Time-lapse microscopy and imaging analysis

Cells were plated into 2-well coverglass bottom chamber slides (Thermofisher Scientific, Waltham, MA, USA) at a density of 20,000 cells per well and allowed to adhere for a minimum of 24 hours at 37°C and 5% CO_2_. Before acquisition, the slides were moved to a Pecon Heating Insert (Carl Zeiss) maintained at 37°C and 5% CO_2_. Time-lapse videos were acquired using a 20X air objective with a CMOS camera (Orca Flash V4.0, Hamamatsu Photonics, Hamamatsu City, Japan) on a Zeiss AxioObserver.Z1 wide-field epifluorescence microscope equipped with an automated stage and focus (Carl Zeiss, Oberkochen, Germany). For the mitotic progression experiments, cells were imaged at 10 minute intervals for 18 hours. Hoechst 33342 (TheromoFisher Scientific, Waltham, MA, USA), a membrane-permeant fluorophore, was carefully added into the imaging medium two hours prior to the completion of the time- lapse experiment, where the cells were then imaged for Hoechst 33342 fluorescence. An algorithm coded in MATLAB (The MathWorks, Natick, MA, USA) was constructed to quantify the integrated nuclear fluorescence of Hoechst 33342 in individual cells. From this algorithm, a cell cycle histogram is generated from all cells imaged in a single field of view, allowing DNA content to be estimated. The ploidy of each mitotic cell was manually determined by summing the amount of DNA content observed in the respective daughter cells. For the FUCCI sensor (ThermoFisher Scientific, Waltham, MA, USA) experiments, cells were co-transduced with 80 particles per cell (PPC) Premo geminin-GFP (G2/M reagent) and 80 PPC Premo Cdt1-RFP (G1/S reagent) for 24 hours. The cells were then washed, released into complete media and imaged 12 hours later at 20 minute intervals for 18 hours. Individual cells expressing both vectors were scored for completion of mitosis, an abortive mitosis or mitotic bypass.

### TCGA analysis of polyploid tumors

Ploidy estimated tumor data generated from TCGA Affymetrix SNP6.0 arrays (as presented in Dewhurst et al.) were kindly provided to our laboratory by Nicolas McGranahan and Charles Swanton, Cancer Research UK London Research Institute, London, UK. These data were then matched with Illumina mRNA sequencing data, excluding samples lacking both ploidy estimations and mRNA sequencing. Z-scores from RSEM normalized mRNA data were then compared between tumor samples estimated to be diploid and polyploid.

### Statistical Analysis

Statistical comparisons were performed using Welch's t-test. P < 0.05 was considered significant. All statistical tests were carried out using the R statistical software, version 3.3.2.

## SUPPLEMENTARY FIGURES



## Abbreviations

14-3-3γ: 14-3-3 gamma
TCGA: The Cancer Genome Atlas
CIN: chromosomal instability
NSCLC: non-small cell lung cancer
FUCCI: fluorescence ubiquitination cell cycle indicators
GFP: green fluorescent protein
FACS: fluorescence activated cell sorting
HRP: horseradish peroxidase
SDS-PAGE: sodium dodecyl sulfate polyacrylamide gel electrophoresis
BCA: bicinchoninic acid assay
TBS-t: tris-buffered saline with tween 20
BSA: bovine serum albumin
NFDM: non-fat dry milk
PVDF: polyvinylidene difluoride
FISH: fluorescence *in situ* hybridization
