# Supplementary material for: The induction of endoreduplication and polyploidy by elevated expression of 14-3-3γ
Source: Genes Cancer. 2017 Nov;8(11-12):771–83. doi: 10.18632/genesandcancer.161 (PMC5755723; doi:10.18632/genesandcancer.161)
Supplement: Supplementary file 1 [file ganc-08-771-s001.pdf]

The induction of endoreduplication and polyploidy by elevated expression of 14-3-3γ - Gomes et al

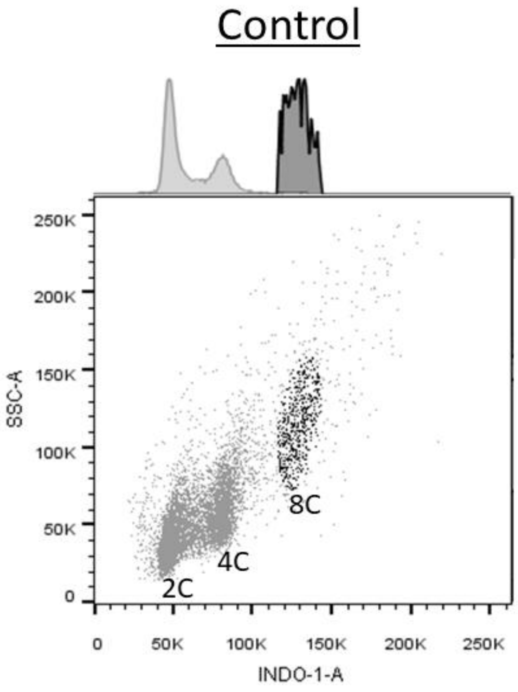

Supplemental figure 1: Sorting of polyploid cells.
Cells were stained with Hoechst 33342. Individual polyploid cells with approximately 8C DNA content (Dark grey) were sorted from the control population (left) and from H322γ cells (right) into individual wells of a 96-well plate with approximately 99% purity. Cell aggregates were gated out of the analysis, determined by Indo-A and Indo-W.

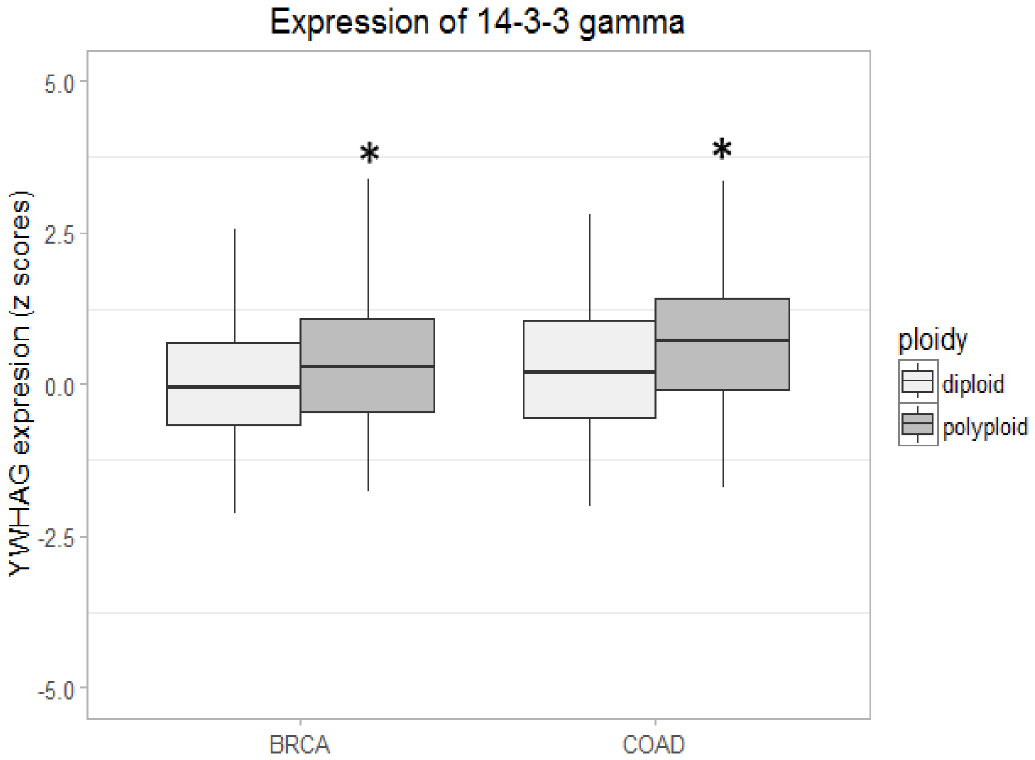

Supplemental figure 2: 14-3-3γ mRNA expression is elevated in breast and colorectal tumors predicted to be genome doubled.

TCGA's Illumina mRNA RSEM normalized z-scores were evaluated in tumor samples predicted to be diploid (light gray) or polyploid (dark gray). Expression of the 14-3-3γ gene (YWHAG) was evaluated was compared against diploid and polyploid samples. Welch's t-test was performed and statistical significance was measured at $p < 0.05$, indicated by an asterisk. [COAD= colorectal adenocarcinoma (n=183), BRCA = Breast carcinoma (n=903)].
